# Kinesin Spindle Protein (KIF11) in Mitosis and Cancer

**DOI:** 10.3390/ijms26188975

**Published:** 2025-09-15

**Authors:** João P. N. Silva, Patrícia M. A. Silva, Hassan Bousbaa

**Affiliations:** 1 UNIPRO—Oral Pathology and Rehabilitation Research Unit, University Institute of Health Sciences (IUCS), Cooperativa de Ensino Superior Politécnico e Universitário (CESPU), 4585-116 Gandra, Portugal; joaosilva_06@hotmail.com; 2 Associate Laboratory i4HB—Institute for Health and Bioeconomy, University Institute of Health Sciences—CESPU, 4585-116 Gandra, Portugal; 3 UCIBIO—Applied Molecular Biosciences Unit, Translational Toxicology Research Laboratory, University Institute of Health Sciences (1H-TOXRUN, IUCS-CESPU), 4585-116 Gandra, Portugal

**Keywords:** KSP, KIF11, molecular motor, mitosis, cancer, cancer therapy

## Abstract

Kinesin spindle protein (KSP), also known as KIF11, is a member of the kinesin superfamily of motor proteins that plays a pivotal role in mitosis by regulating spindle assembly, chromosome alignment, and segregation. Its motor activity, which is essential for the proper organization of microtubules during mitosis, is crucial for maintaining genomic stability. KSP overexpression has been observed in several cancer types, where it promotes uncontrolled cell proliferation, making it a promising target for cancer therapy. This review provides a comprehensive analysis of the molecular mechanisms underlying KSP function, including its structural features, ATPase activity, and interactions with other mitotic proteins. Additionally, we review the regulation of KSP through post-translational modifications, such as phosphorylation, as well as the therapeutic strategies currently being explored to inhibit its activity in cancer treatment.

## 1. Introduction

Kinesin spindle protein (KSP), also known as kinesin family member (KIF) 11 or Eg5, is a plus-end directed kinesin motor protein that plays a central role in microtubule dynamics during mitosis and in other essential cellular processes [1,2,3]. At mitosis, KSP is responsible for the formation and maintenance of bipolar spindles by crosslinking and sliding antiparallel microtubules, which is critical for centrosome separation, chromosome alignment, and successful mitotic progression. Inhibition of KSP disrupts spindle bipolarity, leading to the formation of monopolar spindles and resulting in mitotic arrest and apoptotic cell death in rapidly dividing cells. Recent studies have highlighted the relevance of KSP not only as a mitotic motor but also as a potential therapeutic target in oncology. Overexpression of KSP has been reported in several human cancers, including breast, liver, and lung cancers, and is frequently associated with enhanced tumor proliferation, chemoresistance, and poor prognosis [4,5,6,7].

This overexpression reinforces its role as a driver of genomic instability and a marker of aggressive tumor behavior. In addition to its mitotic function, KSP is also involved in other non-mitotic cellular processes, such as axonal transport, intracellular vesicle trafficking, and microtubule organization in differentiated cells. Its activity is tightly regulated by phosphorylation, particularly by mitotic kinases such as cyclin-dependent kinase (CDK) 1, which control its localization and motor activity throughout the cell cycle [3]. Dysregulation of these pathways can contribute to aneuploidy and oncogenesis. Given its selective activity in proliferating cells and limited expression in non-dividing tissues, KSP has emerged as an attractive target for anticancer therapy. Several small-molecule inhibitors have been developed and have shown promising preclinical activity and have been evaluated in clinical trials for both solid tumors and hematologic malignancies [8,9,10]. While some clinical responses have been modest, especially as monotherapy, combination strategies and biomarker-driven approaches may improve therapeutic efficacy. This review aims to provide a comprehensive overview of KSP’s molecular functions and regulatory mechanisms, including its structural features, interaction partners, and post-translational modifications. Furthermore, we will discuss current progress in the development of KSP inhibitors, their therapeutic potential, and the challenges associated with targeting mitotic motors in cancer.

## 2. Gene and Protein Regulation of KSP

**Figure 1:**
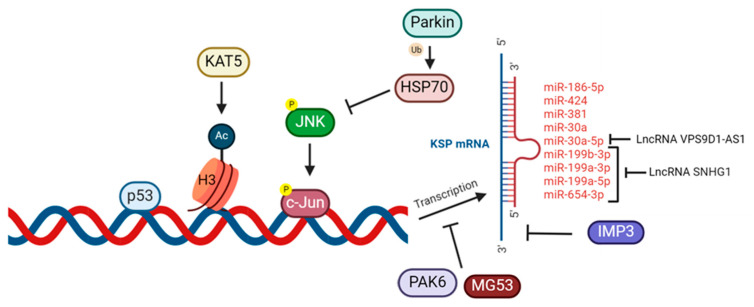
Kinesin spindle protein transcriptional and post-transcriptional regulation. In cancer mutation of p53 and acetylation of histone 3 mediated by KAT5 have been shown to increase KSP gene transcription. On the other hand, Parkin promotes HSP70 ubiquitination (Ub) leading to c-Jun and consequently KSP transcription repression. Moreover, PAK6 and MG53 have also been shown to repress KSP transcription. At the post-transcriptional level several miRNA have been shown to bind to KSP mRNA inhibiting its translation. Nonetheless, long non-coding RNAs have been described to bind some of these miRNAs blocking their binding to KSP mRNA. IMP3 has also been reported to inhibit KSP translation. Abbreviations: H3, histone 3; HSP70, heat shock protein 70; IMP3, insulin-like growth factor-II messenger RNA-binding protein-3; JNK, c-Jun N-terminal kinase; KAT5, lysine acetyltransferase 5; KSP, kinesin spindle protein; LncRNA, long non-coding RNAs; MG53, mitsugumin 53; miR, microRNA; mRNA, messenger RNA; PAK6, p21-activated kinase 6. Created with BioRender.com.

The human KSP gene, *KIF11*, is located at q23.33 on chromosome 10; however its transcriptional regulation has only been described in cancer cells (Figure 1) [11]. For instance, the upregulation of KIF11 in cancer may occur through the mutation of p53, a tumor suppressor protein, or by increased acetylation of histone 3 at lysine (Lys) 27 by lysine acetyltransferase 5 [11]. KIF11 repression can occur via parkin, an E3 ligase, which represses c-Jun N-terminal kinase by promoting heat shock protein 70 (HSP70)-mediated ubiquitination [12]. Post-transcriptionally, LncRNA, like small nucleolar RNA host gene 1, and proteins, such as insulin-like growth factor-2 mRNA-binding protein 3, are involved in the regulation of the expression of KSP in different types of cancer [11,13,14]. Several microRNAs have also been shown to regulate KSP mRNA expression in cancer: miR-186-5p, miR-424, miR-381, miR-30a, miR-30a-5p [11]. Their regulation of KSP expression will be further explored later in this review.

**Figure 2:**
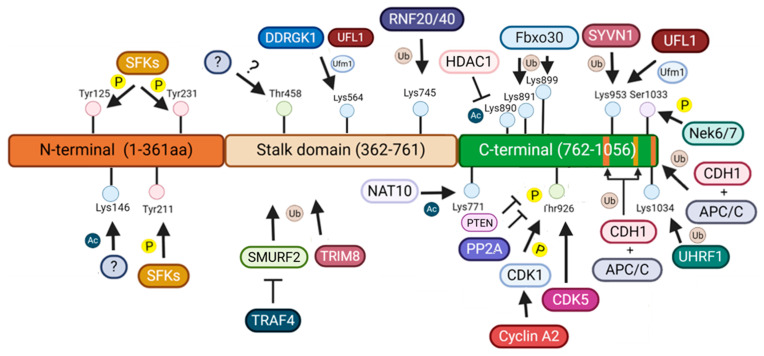
Kinesin spindle protein post-translational regulation. KSP is highly regulated since it has several functions during mitosis and also outside mitosis that need to be timely regulated. Thus, it presents several residues that can be phosphorylated (P), acetylated (Ac), ubiquitinated (Ub) or UFMylated activating or repressing KSP activity or promoting KSP localization to specific parts of the cell. KSP has also KEN box sequence (yellow bar in the C-terminal domain) located at amino acids 1022–1024 and two Destruction box (D-box) sequences (orange bars in the C-terminal domain) at positions 944–947 and 1047–1050 which can be ubiquitinated by CDH1-APC/C leading to KSP degradation. ? refers to unknown proteins and or post-translational modification. Abbreviations: APC/C, anaphase promoting complex/cyclosome; CDH1, CDC20-homolog 1; CDK, cyclin-dependent kinase; FBXO30, F-box only protein 30; HDAC1, histone deacetylase 1; Lys, lysine; NAT10, N-acetyltransferase 10; NEK, NIMA-related kinase; PP2A, protein phosphatase 2A; PTEN, phosphatase and tensin homolog; RNF, RING Finger; Ser, serine; SFKs, Src family kinases; Smurf2, SMAD-specific E3 ubiquitin protein ligase 2; SYVN1, synoviolin 1; Thr, threonine; TRAF4, tumor necrosis factor-receptor associated factor 4; TRIM8, tripartite motif containing 8); Tyr, tyrosine; UFL1, UFM1 specific ligase 1; Ufm1, ubiquitin-fold modifier 1; UHRF1, ubiquitin-like with PHD and RING finger domains 1. Created with BioRender.com.

The KSP is constituted by three domains: a tail domain (C-terminal), a motor domain (N-terminal) and a central stalk domain (Figure 2) [15].

### 2.1. Tail Domain

During mitosis, the tail domain enhances the motor domain affinity to microtubules and plays a crucial role for directing KSP to spindle microtubules [16]. The phosphorylation of the threonine (Thr) 926 residue in this domain during prophase, mediated by the CDK1, is essential for the interaction of KSP with ERCC2/XPD, a subunit of transcription factor II H (Table 1). This interaction promotes the localization and association of KSP to microtubules during metaphase, contributing to bipolar spindle formation [3,17,18,19,20,21]. In neurons, the phosphorylation of KSP at Thr926 is mediated by CDK5 instead of CDK1 [22]. Phosphatase and tensin homolog (PTEN) regulates the phosphorylation levels of KSP at Thr926 preventing KSP hyperphosphorylation and consequently mitotic spindle defects [23]. Moreover, the dephosphorylation of the Thr926 residue performed by protein phosphatase 2A at late metaphase is crucial for mitotic progression [24]. CDK1 also phosphorylates Tiam1, a guanine-nucleotide exchange factor, leading to the activation of p21-activated kinases (PAK) and promotion of chromosome congregation by counteracting KSP during bipolar spindle assembly [25,26].

**Table 1:** Post-translational modifications of KSP and its functional impacts.

Modification Sites	Interacting Molecules	Modification	Functional Impacts	References
Tyr125	SFKs	Phosphorylation	Essential for complete motor activity	[27]
Lys146	Unknown	Acetylation	Enhances motor performance	[28]
Tyr 211	SFKs	Phosphorylation	Essential for complete motor activity	[27]
Tyr 231	SFKs	Phosphorylation	Essential for complete motor activity	[27]
Thr458	Unknown	Phosphorylation	Unknown	[17]
Lys564	UFL1 and DDRGK1	UFMylation	Promotes spindle localization	[29]
Lys745	RNF20/40	Ubiquitination	Promotes KSP stabilization	[30]
Lys771	NAT10	Acetylation	Stabilizes KSP and promotes its localization to centrosomes	[31]
Lys890	HDAC1	Deacetylation	Activates KSP ATPase activity	[32]
Lys891	FBXO30	Ubiquitination	Essential for mammopoiesis regulation	[11,33]
Lys899	FBXO30	Ubiquitination	Essential for mammopoiesis regulation	[11,33]
Thr926	CDK1	Phosphorylation	Promotes the localization and association of KSP to microtubules	[3,17,18,19,20,21]
	CDK5	Phosphorylation	Occurs only in neurons and promotes the localization and association of KSP to microtubules	[22]
	PTEN	Dephosphorylation	Prevents KSP hyperphosphorylation	[23]
	PP2A	Dephosphorylation	Crucial for mitotic progression	[24]
Lys953	SYVN1	Ubiquitination	Promotes KSP degradation	[34]
	UFL1	UFMylation	Prevents KSP ubiquitination mediated by SYVN1 and consequently its degradation	[34]
Ser1033	NEK6/7	Phosphorylation	Promotes KSP centrosome localization and motor function	[19,35]
Lys1034	UHRF1	Ubiquitination	Promotes KSP interaction with TPX2 and consequently, centrosome localization	[36]
?	TRIM8	Ubiquitination (?)	Essential for bipolar spindle formation and cell cycle progression	[37]
	SMURF2	Ubiquitination	Promotes KSP degradation	[38]
KEN box	CDH1-APC/C	Ubiquitination	Promotes KSP degradation	[39]
D-box				

? refers to unknown modification site or uncertain post-translational modification.

The phosphorylation of the residue serine (Ser) 1033 in the tail domain by NIMA related kinase (NEK) 6/NEK7 is essential for KSP centrosome localization, motor function and consequently for the normal mitotic progression. Ser1033 phosphorylation is needed for the interaction between KSP and the targeting protein for Xklp2 (TPX2) and decreases the interaction between KSP and XPD [19,35]. The phosphorylation of both KSP and TPX2 is regulated by NEK9 kinase, which in turn is regulated by CDK1 and polo-like kinase 1 (PLK1). NEK9 is involved in the phosphorylation of KSP through NEK6/NEK7 activation and phosphorylates TPX2 directly. The phosphorylation of TPX2 is needed for its localization at the centrosomes during prophase [35]. Phosphorylation of KSP at Ser1033 also appears essential for the formation of a complex with disks large homolog 1 which is crucial for the interaction with PTEN. PTEN is phosphorylated by PLK1 leading to its recruitment to centrosomes where it then interacts with KSP-disks large homolog 1 also promoting KSP localization to the centrosomes [40]. However, phosphorylation of Ser1033 was shown to occur only in a small fraction of the total spindle associated KSP suggesting the existence of different pools of KSP that perform different functions [17,41].

PLK1 and PTEN are also involved in the regulation of valosin-containing protein (VCP) by phosphorylating and dephosphorylating VCP, respectively. The phosphorylation of VCP occurs at the centrosome, while the PTEN-mediated dephosphorylation is essential for the presence of both VCP and KSP at the spindle. This regulation promotes the correct segregation of chromosomes [42].

An additional regulatory site of KSP essential for its interaction with TPX2 is the Lys1034 residue which is the substrate of ubiquitin-like with PHD and RING finger domains 1. Ubiquitin-like with PHD and RING finger domains 1 is a cell cycle regulator and epigenetic coordinator that, during metaphase, interacts with KSP polyubiquitinating it and promoting KSP interaction with TPX2 [36].

The tail domain can also be UFMylated in the Lys953 residue by ubiquitin-fold modifier 1-specific ligase 1 which competes with Synoviolin 1 preventing it from ubiquitinating KSP and inhibiting KSP degradation [34]. Tripartite motif (TRIM) 8, an E3 ligase, also interacts with KSP which is essential for bipolar spindle formation and cell cycle progression. It is suggested that TRIM8 ubiquitinates KSP but the mechanism of regulation needs to be further investigated [37]. In addition, ubiquitination of Lys891 and Lys 899 by F-box protein 30, an E3 ligase, is essential for mammopoiesis regulation [11,33]. Furthermore, CDC20-homolog 1 (CDH1) in association with the anaphase promoting complex/cyclosome (APC/C) promotes KSP ubiquitination and consequent degradation. Several sites of ubiquitination mediated by CDH1-APC/C have been found in KSP. These include a KEN box sequence located at amino acids 1022–1024 and two D-box sequences at positions 944–947 and 1047–1050 [39].

KSP acetylation and deacetylation regulate its stability and motor activity during mitosis. The nucleolar acetyltransferase N-acetyltransferase 10 acetylates Lys771, stabilizing KSP and localizing it to centrosomes for proper chromosome segregation [31]. Lys890 is deacetylated by HDAC1 during prophase, activating KSP ATPase activity, while deacetylation of Lys146 enhances motor performance under load and strengthens the neck linker–catalytic site connection [28,32].

### 2.2. Motor Domain

KSP motor domain was shown to stabilize microtubules at the plus-end tip and to promote microtubule nucleation [43]. Phosphorylation of the tyrosine (Tyr) 125, Tyr211, and Tyr231 residues, in the motor domain, by SRC family kinases, is necessary for a complete motor activity. In vitro, mutations in these residues showed that the Tyr211 mutation led to the biggest motor activity changes, while inhibition of Tyr211 phosphorylation led to increased spindle defects including shorter and fragmented poles, and monopolar spindle phenotype [27].

### 2.3. Stalk Domain

In human cells, the Thr458 residue in the stalk domain was shown to be a possible site of phosphorylation of two proteins involved in DNA damage response [17]. Furthermore, the residue Lys564 is UFMylated probably by interaction with ubiquitin-fold modifier 1-specific ligase 1 and DDRGK1 promoting KSP spindle localization. Prevention of Lys564 UFMylation leads to mitotic arrest, shorter spindles and inhibits cell division [29].

## 3. Cell Cycle-Dependent Localization and Functional Dynamics of KSP

KSP functions in different stages of the cell cycle and thus its localization changes according to the cell cycle phase the cell is undergoing. During interphase KSP is located in the cytoplasm, while in prophase, it localizes at the centrosome and spindle pole. In metaphase, KSP plus-end-directed motility directs it to the spindle microtubules whereas in anaphase it relocates to the spindle midzone [11].

**Figure 3:**
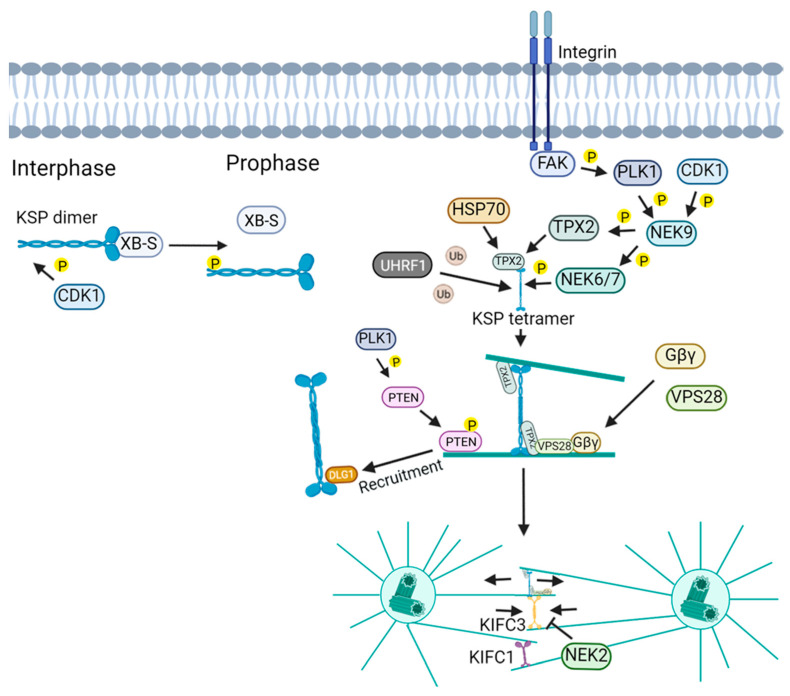
Schematic representation of centrosomes migration to the poles. In interphase, inactive KSP is bound to XB-S. In prophase, KSP is phosphorylated dissociating from XB-S. Integrin activates FAK which phosphorylates PLK1 which alongside CDK1 phosphorylates NEK9 activating it. NEK9 phosphorylates TPX2 and NEK6/7. PLK1 also phosphorylates PTEN promoting its localization at the centrosomes where it interacts with the complex KSP-DLG1 recruiting it to the centrosome. HSP70 promotes the association of the phosphorylated TPX2 with KSP which will then promote KSP localization to microtubules. UHRF1, Gβγ and VPS28 also promote the localization of the complex KSP-TPX2 to microtubules. NEK 6/7 phosphorylates KSP activating its motility function. Active KSP can then produce outward forces separating centrosomes to opposite poles. KIFC1 and KIFC3 also bind to microtubules exerting opposite forces to KSP which is essential for the proper regulation of centrosomes separation. At late G2, NEK2 inactivates KIFC3 pending the balance in KSP direction leading to centrosome bipolarity. Abbreviations: CDK1, cyclin-dependent kinase 1; DLG1, disks large 1; FAK, focal adhesion kinase; HSP70, heat shock protein 70; KIF, kinesin family member; KSP, kinesin spindle protein; NEK, NIMA related kinase; PLK1, polo-like kinase 1; PTEN, phosphatase and tensin homolog; TPX2, targeting protein for Xklp2; UHRF1, ubiquitin-like, containing PHD and RING finger domains 1; VPS28, vacuolar protein sorting-associated protein 28. Created with BioRender.com.

During interphase, KSP remains largely inactive through association with XB-S, forming a reservoir that can be released upon phosphorylation when needed for mitosis (Figure 3) [44]. In prophase, centrosome separation is driven by two complementary pathways: a KSP-dependent mechanism, based on the sliding of antiparallel microtubules by KSP homotetramers, and a dynein-dependent pathway [45].

The function of KSP in microtubule motility depends on the formation of a homotetramer. KSP activity is further modulated by focal adhesion kinase (FAK) signaling, counteracting minus-end-directed kinesins (KIFC1/KIFC3), and centrosome-associated regulators such as hematological and neurological expressed 1, PLK1, and Aurora A [46,47,48]. Additionally, hematological and neurological expressed 1, with γ-tubulin in centrosomes, is suggested to play an important role in centrosome maturation and formation of the mitotic spindle by promoting the proper localization of KSP at centrosomes, the association of PLK1 and Aurora A and stability of PCM1-y-tubulin binding [49]. These coordinated interactions ensure proper spindle assembly and bipolarity, while defects in this balance can be partially compensated by dynein activity, although at the cost of increased segregation errors [50,51].

**Figure 4:**
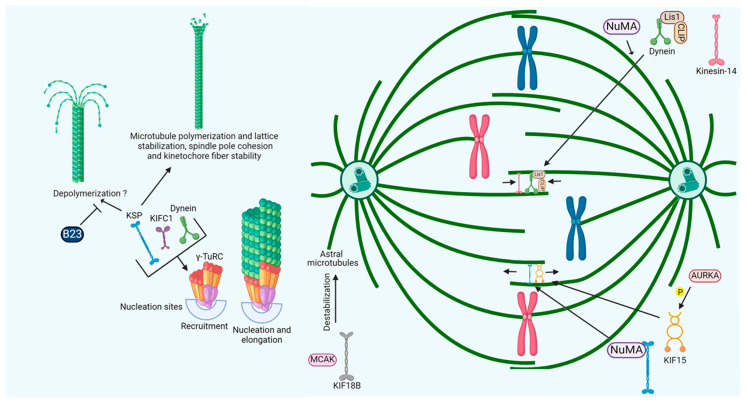
KSP functions and localization during metaphase. KSP, KIFC1 and Dynein are involved in microtubule nucleation. While KSP promotes polymerization and lattice stabilization, spindle pole cohesion and kinetochore fiber stability. It has also been described that KSP promotes depolymerization and that B23 inhibits this function. During metaphase opposing forces are in play to allow microtubules to properly attach to chromosomes and to guide chromosomes to the mitotic plate. NUMA promotes dynein associated with Lis1 and Clip-170 localization at the mitotic spindle where it exerts inward force. Kinesin-14 has also been shown to exert inward forces. On the contrary KSP and KIF15, after Aurora A phosphorylation, produce outward forces promoting spindle bipolarity maintenance. Abbreviations: γTuRCs, γ-tubulin ring complexes; AURKA, aurora kinase A; CLIP-170, cytoplasmic linker protein 170; KIF, kinesin family member; KSP, kinesin spindle protein; Lis1, lissencephaly-1; MCAK, mitotic centromere-associated kinesin; NuMA, nuclear mitotic apparatus protein. Created with BioRender.com.

During prometaphase and metaphase, KSP interacts with TPX2, HSP70, and vacuolar protein sorting-associated protein 28 to localize to spindle microtubules and coordinate with dynein for pole-directed transport [41,52,53,54,55]. KSP, along with dynein, kinesins, and KIF15, drives spindle assembly and maintains bipolarity through opposing forces, regulated by CDK1 and Aurora kinases [56,57,58,59,60,61,62]. NuMA recruits KSP, astrin, and dynein to stabilize spindle poles, while KSP crosslinks microtubules near poles to maintain cohesion (Figure 4) [63,64,65,66]. KSP also promotes microtubule polymerization, nucleation, and lattice stabilization, becoming critical for bipolarity when KIF2C is inhibited [67].

During anaphase, mitotic spindle needs to elongate for correct chromosome segregation. KIF4A and KSP drive this process by sliding antiparallel microtubules, generating the necessary force, while their inhibition blocks elongation and causes segregation defects [68]. Microtubules are bundled by PRC1, regulated by CDK1-cyclin B, ensuring proper crosslinking. Aurora B phosphorylates KIF4A, activating its ATPase, promoting its association with PRC1, and repressing microtubule growth [69,70]. Proper kinetochore–microtubule attachments move out of the Aurora B gradient, while merotelic attachments causing lagging chromosomes can be corrected. KSP inhibition reduces spindle elongation and lagging chromosomes. After metaphase, KSP regulation by APC/C–CDH1 prevents spindle multipolarity and supports centrosome clustering in the next metaphase [71,72].

## 4. Other Functions of KSP

In addition to the functions described above, other roles for KSP have been described. For instance, KSP is essential for proper mammopoiesis [33], spermatogenesis [73], early embryonic development [74,75], oocyte function [76], protein traffic [77], polypeptide synthesis [2], osteogenesis [78]. It is also involved in neuronal function and diseases [79,80,81,82,83], Alzheimer’s disease [84,85], ciliary function [86], familial exudative vitreoretinopathy, a retinal disease which causes blindness [87,88,89], and other diseases such as rheumatoid arthritis [90] and HPV infection [91].

## 5. KSP in Cancer

In cancer, KSP is frequently deregulated, contributing to chromosomal instability, uncontrolled cell proliferation, tumor progression, and metastasis. The overexpression of KSP has been consistently observed across multiple cancer types, as demonstrated by proteomic data analysis using the UALCAN webtool. This deregulation is often linked to poor clinical outcomes. This section explores the multifaceted roles of KSP in cancer biology, including its impact on chromosome stability, tumor growth, metastatic potential, and drug responses.

Cancer cells are characterized by their chromosomal instability which can promote aneuploidy. Tetraploidy is suggested to be an intermediate state before the cell turns aneuploid and recently it was reported that the level of functional KSP controls this transformation. For instance, it was reported that high levels of functional KSP causes multipolarity which generates aneuploid cells after cell division, while low levels of functional KSP maintain bipolarity and daughter cells persist as tetraploid cells [92]. In addition, following whole-genome duplication, tetraploid cells where KSP was partial depleted underwent bipolar mitosis instead of remaining multipolar [93]. Recently, it was shown that even a modest upregulation of KSP expression can cause mitotic defects leading to chromosome instability [94]. Therefore, overexpression of KSP seems to promote multipolarity and consequently chromosomal instability. Chromosomal instability can lead to tumor development and, also, to resistance to treatment, which is advantageous to cancer cells.

KSP has also been implicated in cancer cells proliferation and carcinogenesis. For instance, KSP promotes proliferation in gallbladder cancer via the HER2/PI3K/AKT signaling pathway [95]. Moreover, PAK6 regulates proliferation and progression in hepatocellular carcinoma (HCC) by negatively regulating KSP expression [96]. High interstitial fluid pressure has also been implicated in the progression of HCC possibly through the promotion of ubiquitin-specific peptidase 1 activity. Ubiquitin-specific peptidase 1 deubiquinates the residue Lys77 of KSP preventing its degradation, consequently promoting HCC progression [97]. KSP also induces HCC progression by promoting octamer-binding transcription factor 4, which plays an essential role in differentiation, and through the Wnt/β-catenin pathway by associating with assembly factor for spindle microtubules [98,99]. In addition, Di-(2-ethylhexyl) phthalate, an endocrine-disrupting chemical which can disrupt essential biological processes in the human body, has been shown to induce benign prostatic hyperplasia by promoting the KSP-mediated activation of the Wnt/β-catenin pathway [100]. In breast cancer (BC), the activation of this pathway by KSP increases cells self-renewal [101]. KIF11 is overexpressed in colorectal cancer (CRC) cells when compared with normal cells suggesting a critical role of KSP in tumor development and progression [102].

In BC, the miRNA miR-30a was shown to negatively regulate KSP, while KSP knockdown hindered proliferation and invasion. Consistently, lower levels of miR-30a and high levels of KSP mRNA were associated with poor prognosis [103].

On the other hand, malignant progression of lung adenocarcinoma (LUAD) is induced by the overexpression of the LncRNA VPS9D1-AS1 which upregulates KSP by interacting with miR-30a-5p [104]. In neuroblastoma (NB) cells, miR-186-5p downregulates KSP inhibiting cell growth. In addition, in tumor tissue, miR-186-5p was underexpressed when compared to tumor adjacent tissues, while the inverse was observed for KSP [105]. In BC, tumor necrosis factor receptor-associated factor 4 was shown to inhibit SMAD-specific E3 ubiquitin protein ligase family member 2 and consequently the ubiquitination of KSP repressing apoptosis and inducing proliferation [38]. Furthermore, Mitsugumin 53, a protein part of the TRIM family, was shown to repress KSP transcription in pancreatic ductal adenocarcinoma inhibiting tumor growth [106]. The ubiquitin ligase RNF20/40 monoubiquitinates KSP at Lys745, stabilizing it. The RNF20/40-KSP axis is implicated in breast carcinogenesis [30].

In LUAD, KSP mRNA expression is associated with tumorigenesis and cancer development while it also correlates with higher immune infiltration [107]. Moreover, KSP in association with helicase lymphoid-specific was shown to induce LUAD progression by promoting AKT and cAMP response element binding-protein phosphorylation [108]. Similarly, in non-small cell lung cancer and small-cell lung cancer KSP is also implicated in cancer progression and proliferation [109,110].

Moreover, in anaplastic thyroid carcinoma, lysine acetyltransferase 5 is overexpressed and promotes the proliferative activity of cancer cells while repressing apoptosis and the induction of autophagy by inducing KSP expression [111].

In TP53 mutant glioma, chemoresistance, cell proliferation and stemness were promoted by KSP upregulation of cyclins expression [112]. Vascular endothelial growth factor (VEGF)-A signaling was also shown to promote cell proliferation by inducing several kinesins, including KSP, activity, while the inhibition of KSP with Ispinesib or Dimethylenastron impaired angiogenesis both in vitro and in vivo [113]. KSP is essential for mitosis and is involved in several pathways that promote cell proliferation, progression and carcinogenesis. Not surprisingly, KSP upregulation increases cell proliferation and contributes to tumor progression and carcinogenesis in several cancer types.

In addition, KSP plays a crucial role in metastasis in several cancers. In colon cancer cells, cyclin A2 regulates the establishment of symmetrical bipolar spindle and centrosome amplification by promoting CDK1-mediated KSP phosphorylation at the residue Thr926 [114]. Furthermore, overexpression of the NEK9-KSP axis correlates with distant metastasis [115]. In glioblastoma (GBM), KSP has been implicated in cell proliferation and invasion [116]. For instance, it was shown that phosphofructokinase-1 muscle isoform, involved in glycolysis regulation, interacts with KSP in the cytosol regulating invasion and cell cycle [117]. Lung cancer cells expressing EML4–ALK V3 have higher rates of metastasis. EML4-ALK V3 forms a complex with NEK9 and NEK7 promoting a mesenchymal-like morphology. NEK7 then phosphorylates KSP inducing its motor activity which was shown to be required for the change in morphology in lung cancer cells [118]. In pancreatic ductal adenocarcinoma, KSP stabilizes SREBP2, a transcription factor, inhibiting its ubiquitination. Conversely, it was also proposed that KSP proliferation and migration promotion was dependent on SREBP2 activity [119]. Nonetheless, KIF11 knockdown led to the impairment of LUAD migration and invasiveness, while KIF11 knockdown in glioma cells reduced the PAK1-mediated cell migration [4,120]. Furthermore in ovarian cancer, death receptor 6, involved in the mediation of cell apoptosis and immune response, was shown to promote migration via association with TRAF4 and KSP [121]. In BC, KSP was shown to respond to EGF-mediated chemotaxis, regulating the direction of cell migration [122]. Additionally, KSP expression is associated with increased spindle length and consequently metastatic cells. It is suggested that the increased spindle length occurs due to the pushing force, essential for the separation of the two spindle poles, created by KSP [123]. It is also suggested that in esophageal squamous cell carcinoma, KSP is important for cell proliferation and migration [124].

KSP is involved in proliferation and invasion which are important for the metastatic process. Accordingly, in several types of cancer, KSP leads to an increase in metastasis, which is associated with tumor aggressiveness. Thus, cancer patients’ KSP expression might be an indicator of disease progression and potential aggressiveness.

### 5.1. KSP Overexpression in Cancer Across CPTAC Datasets Using UALCAN Analysis

Using the UALCAN webtool to assess KSP level in cancer when compared to normal samples it is possible to observe that KSP is overexpressed in almost all cancer types with available data, with the exception of clear cell renal cell carcinoma (Table 2). This upregulation has also implication in clinical outcomes since the overexpression of KSP is associated with a poor prognosis in LUAD [4,125], triple negative breast cancer (TNBC) [126,127], BC [5,128], laryngeal squamous cell carcinoma [129], endometrial cancer [130], HCC [6,7,131,132,133,134,135], esophageal squamous cell carcinoma [124], Wilms’ tumor [136], astrocytoma [137], bladder urothelial carcinoma [138], renal cell carcinoma [139,140,141], CRC [142], NB [143,144], cholangiocarcinoma [145], gastric cancer [146] and meningioma [147]. Particularly, in oral cancer, through immunohistochemical analysis it was shown that tumor tissues expressed KSP, while normal oral epithelia tissues did not. In addition, high expression of KSP is associated with poor prognosis in oral cancer patients [148]. In prostate cancer, higher KIF11 expression is associated with worse disease-free survival and metastasis-free survival [149]. High expression of both KSP and inhibitor of differentiation (ID) 1 genes were associated with worse relapse-free survival in BC patients [150]. Moreover, in pancreatic cancer, high expression of KSP was associated with worse postoperative prognosis [151]. In endometrioid carcinoma, high expression of KIF11 and low expression of KIF14 independently were associated with poor prognosis. Interestingly, patients showing both high KIF11 and low KIF14 expression were associated with the worst prognosis for this type of cancer [152].

### 5.2. Kinesin Spindle Protein Effects on Drug Responses

KSP expression has also been reported to be associated with some drugs response.

For instance, in non-small cell lung cancer, KSP expression was positively correlated with patient response to treatment with Cisplatin, an alkylating agent, plus Vinorelbine or Docetaxel or with Carboplatin and Paclitaxel [153]. However, higher expression of KSP was observed in Cisplatin resistant lung cancer samples [110]. Moreover, in prostate cancer, KSP nuclear expression has been correlated with aggressiveness and loss of KSP nuclear expression seems to be associated with Docetaxel resistance [154]. Nonetheless, KSP is overexpressed in a stem-like cell subpopulation of Docetaxel-resistant TNBC cells. Cancer stem cells have been associated with chemoresistance and thus it is suggested that KSP plays a role in TNBC Docetaxel resistance [126]. Additionally, downregulation of KSP in HCC inhibited cell proliferation while increasing apoptosis and cell sensitivity to Doxorubicin, a topoisomerase 2 inhibitor [155].

Overexpression of KSP has been mostly reported to confer resistance to microtubule targeting agents in various cancer types. Additionally, the subcellular localization of KSP seems to play a role in modulating drug resistance. However, these findings should not be generalized across all cases, as the expression and impact of KSP should be evaluated on a case-by-case basis to understand its role in patient responses to treatment.

## 6. Targeting KSP in Cancer

Since KSP plays such a critical role in different processes involved in cancer development and progression and it is overexpressed in several tumors, different drugs targeting this protein have been explored mostly aiming cancer treatment. KSP inhibition leads to mitotic arrest at G2/M phase through spindle assembly checkpoint (SAC) activation [15]. SAC is a checkpoint mechanism that prevents mitotic progression due to the impairment of the spindle assembly machinery. This allows the cell to correct errors and prevent chromosome missegregation and consequently aneuploidy. However, if the cell remains in mitotic arrest for a prolonged period, programmed cell death is induced leading to cell death [156]. One advantage of KSP inhibition is that it does not target microtubules which theoretically can decrease cancer therapy toxicity since antimitotics such as Paclitaxel and Docetaxel are widely used in cancer treatment [157]. Moreover, in animal models, KSP inhibition prompted immune responses since increased number of lymphocytes were observed in the spleen and the blood after KSP inhibition [15].

KSP inhibitors can be categorized in two groups according to their binding sites: L5 loop binding allosteric inhibitors and ATP binding competitive inhibitors [20]. The first group of inhibitors binds to a specific site in the motor domain of KSP close to the ATP binding site which triggers a loop L5 rearrangement leading to a transition from an open to close state, consequently, slowing the release of ADP and impairing KSP activity. Some inhibitors that are part of this group are monastrol, MK-0731, S-trityl-L-cysteine (STLC), K858, Ispinesib and Filanesib [20,158].

The second group of inhibitors also binds to a specific region close to the ATP binding site, but differently from the first group it prevents ATP or ADP binding completely. GSK-1 and BRD9876 are some inhibitors belonging to this group [20,158].

Moreover, optimization of existing KSP inhibitors, synthesis of new KSP targeting agents and discovery of compounds previously unknown to target KSP have been reported and their therapeutic potential being explored in several cancer cell types [159,160,161,162,163,164].

In the following sections results regarding the effects of these drugs individually or in combination for the treatment of cancer both in vitro and in clinical trials will be described.

### 6.1. KSP Inhibitors as Monotherapy

KSP inhibitors individually have been showed to exert promising antitumoral activity in several types of cancers cells [165,166,167].

Filanesib is a potent KSP inhibitor that has been extensively investigated for the treatment of several types of cancer. For instance, in gastric cancer, Filanesib led to repression of cancer cells proliferation both in vitro and in vivo [146]. Addition of Filanesib as well as other KSP inhibitors (SB743921, Ispinesib or AR649) to NB organoids led to mitotic arrest and induction of cell death. Moreover, Filanesib and AR649 were shown to reduce tumor growth and increase survival time in mice carrying NB patient-derived xenograft [143]. In type I epithelial ovarian cancer cells, Filanesib showed similar antitumoral effects as Paclitaxel, but contrarily to Paclitaxel treatment, it does not promote the nuclear factor-κB (NF-κB) and ERK pathways nor the production of cytokines which are associated with chemoresistance [168]. Hematologic cells are suggested to rely on myeloid cell leukemia 1 (MCL-1), an antiapoptotic protein, to prevent apoptosis. In multiple myeloma (MM) cell lines, treatment with Filanesib caused mitotic arrest inducing apoptosis in cells where MCL-1 degradation occurred. Furthermore, in one of the cell lines used in the study, a delay of apoptosis was observed which correlated with lower levels of MCL-1 degradation. Thus, it is suggested that stabilization of MCL-1 can be a potential process for cancer cells to resist treatment with KSP inhibitors. During mitotic arrest cell fate seems to be determined by the degradation of prosurvival proteins and cyclin B1 degradation. If degradation of prosurvival proteins reaches its threshold first cell death is promoted. On the contrary, if cyclin B1 degradation reaches its threshold first the cell can overcome, in this particular case, KSP inhibition [169]. Furthermore, Filanesib showed anticancer activity in vitro and in several mice xenograft models but particularly in hematological carcinomas [170]. In acute myeloid leukemia, Filanesib leads to mitotic arrest and induces apoptosis [171]. In cholangiocarcinoma, both Filanesib and SB743921 caused mitotic arrest leading to cell death in vitro as well as in vivo [145]. Moreover, treatment with Filanesib or Ispinesib led to meningioma growth inhibition both in vitro and in vivo [172]. Filanesib also led to mitotic arrest and proliferation inhibition in hepatoblastoma cells while reducing tumor growth in vivo [173].

Ispinesib is one of the most studied KSP inhibitors, investigated for its potential as an anticancer therapeutic agent. In pancreatic cancer, Ispinesib reduced cell proliferation and promoted apoptosis in vitro, while it repressed tumor growth in vivo. Moreover, it was shown that KSP expression influences Ispinesib efficacy highlighting the importance of assessing KSP expression before administration of Ispinesib in this type of cancer [151]. In BC, Ispinesib repressed cell proliferation in vitro, while it reduced tumor growth in vivo. Furthermore, in xenografts with the MDA-MB-468 cell line, Ispinesib led to complete regression [174].

STLC and analogs: STLC is a reversible KSP inhibitor part of the L5 loop binding allosteric inhibitors group. Alongside its analogs S-(methoxytrityl)-L-cysteine (S(MeO)TLC) and CF3-STLC, STLC has been explored in preclinical trials to assess its antitumoral efficacy. In Docetaxel-resistant and non-resistant prostate cancer cell lines, STLC was shown to promote arrest in G_2_/M, increasing polyploidy. Nonetheless, in the Docetaxel-resistant cells STLC led to higher number of cells arrested in G_2_/M and less apoptosis than in the non-resistant cells [175]. STLC in in vitro and in vivo prostate cancer models showed anticancer activity by arresting cells in mitosis and inducing cell death [165]. In RCC, S(MeO)TLC and STLC anticancer effects were assessed, and repression of cell proliferation was found to be time-dependent for both inhibitors. Furthermore, S(MeO)TLC showed lower IC_50_ values for all cell lines and exposure times evaluated. In vivo, S(MeO)TLC administration resulted in tumor growth inhibition [176]. In bladder cancer, S(MeO)TLC led to mitotic arrest and apoptosis induction in vitro, while in vivo, it decreased tumor growth and increased mice survival [177]. The STLC analog CF3-STLC, in a chronic myeloid leukemia cell line, led to apoptosis induction. Nonetheless, addition of a caspase inhibitor did not interfere with the induction of apoptosis meaning that CF3-STLC can promote cell death through a pathway that does not involve caspase [178]. In addition, the knockdown of KIF15, disks large-associated protein 5, carnosine N-methyltransferase 1 and sterile alpha motif and HD domain-containing protein 1 were reported to increase sensitivity to STLC treatment [179].

K858 is an ATP-uncompetitive KSP inhibitor that, in GBM cells, demonstrated antiproliferative activity and reversed their malignant invasive phenotype [180]. Similar effects were observed in head and neck squamous cell carcinoma cells [181]. In CRC cells, K858 led to mitotic arrest and induction of cell death, while it reduced tumor growth in a mouse ovarian cancer model. Moreover, some cells were shown to escape mitotic arrest, a process known as mitotic slippage, and became polyploid subsequently undergoing senescence [182]. Addition of K858 or an analog led to inhibition of cell proliferation, induction of apoptosis and decrease in survivin expression in cell lines from melanoma and prostate cancer [183]. In BC, the inhibition of KSP with K858 analogs led to decreased NF-κB and consequently MMP-9 expression. MMP-9 is associated with tumor metastasis and invasion, and its transcription is regulated by NF-κB. In addition, the expression of hypoxia-inducible factor 1 and VEGF was also reduced by KSP inhibition [184]. Similarly, in gastric adenocarcinoma cells, KSP inhibition led to a reduction in VEGF expression suggesting that KSP might be involved in angiogenesis. When K858 inhibitors were combined with Hesperidin, a polyphenol found in citrus fruits, VEGF expression reduction was increased [185]. Nonetheless, K858 and its analogs for the most part did not significantly change MMP-9 expression in gastric adenocarcinoma cells. Nonetheless, these compounds repressed cell migration [186].

SB743921 is a small molecule inhibitor of KSP ATPase which, in breast cancer, promoted mitotic arrest while increasing apoptosis. Moreover, SB643921 led to reduction in the prosurvival protein B-cell lymphoma 2 (BCL-2) and denticleless E3 ubiquitin–protein ligase homolog expression whereas it increased the expression of p53 and caspase-3 [187]. SB743921 in chronic myeloid leukemia also led to mitotic arrest and induction of apoptosis while repressing ERK and AKT activity. It also sensitized chronic myeloid leukemia cells to Imatinib, inhibitor of multiple tyrosine kinases [188]. Furthermore, SB743921 arrested diffuse large B-cell lymphoma cells in G_2_/M and promoted apoptosis [189].

Dimethylenastron: the potent KSP inhibitor Dimethylenastron has been shown to reduce cell proliferation, migration and invasion in pancreatic cells, [190]. While in vivo it reduced tumor growth through induction of apoptosis [191]. Additionally, in pancreatic and lung cancer cells, this inhibitor led to mitotic arrest and increased apoptosis [192].

Moreover, tetraploid CRC cells were shown to be more sensitive to Dimethylenastron than diploid cells. Tetraploid cells had a shorter mitotic arrest and seem to advance to cytokinesis, while diploid cells have longer arrests and reverse from metaphase to interphase [193].

Other KSP-targeting agents: the antitumoral efficacy of several other KSP targeting agents has also been explored and the results will be described in this section. In HCC cell lines, CPUYL064 led to cell arrest at G_2_/M and promoted cell death. Moreover, the anticancer effects were shown to be dose- and time-dependent [194]. Similarly, in CRC cells, CPUYJ039 promoted G_2_/M arrest and induced apoptosis in a dose- and time-dependent manner [195]. MK-0731 has been reported to repress tumor growth in both Paclitaxel resistant and non-resistant cells in vivo [196]. While KSP targeting with LY2523355 led to mitotic arrest and induction of apoptosis both in vitro and in vivo in several cancer types [197]. In a variety of cancer cells, YL001 leads to mitotic arrest and increased apoptosis. In vivo, it reduced tumor growth and improved survival [166]. In ovarian cancer, KPYB10602 causes mitotic arrest and apoptosis induction in vitro and, with little neurotoxicity, in vivo [198]. Moreover, the KSP inhibitor CK0106023 caused mitotic arrest and inhibited proliferation in different cancer cell lines [199]. In human skin and melanoma histocultures, SCH2047069, a KSP inhibitor capable of crossing the blood–brain barrier, impaired cell proliferation [200]. HR22C16 and its analogs inhibited cell proliferation in both Paclitaxel-sensitive and Paclitaxel-resistant ovarian cancer cell lines. The analog HR22C16-A1 was further characterized and found to induce mitotic arrest and promote apoptosis through the intrinsic pathway. Moreover, it showed antagonistic effects when combined with Paclitaxel [201]. In prostate cancer, a KSP siRNA led to mitotic arrest and increased apoptosis in vitro and decreased tumor proliferation in vivo. When combined with Paclitaxel antagonistic effects were observed both in vitro and in vivo [202]. Furthermore, another KSP-specific siRNA was also demonstrated to induce tumor growth reduction in melanoma and ovarian cancer mouse models [203].

### 6.2. Combinatorial Approaches

Besides the administration of KSP inhibitors alone, various combinatory approaches have been explored in different types of cancer. For instance, Ispinesib in combination with Genistein, a isoflavone found in soy, showed enhanced cell growth repression and induction of apoptosis in prostate cancer cells [204]. Furthermore, inhibition of KSP leads to decreased cell proliferation and sensitizes cells to Oxaliplatin, an alkylating agent, in CRC, while it radiosensitizes GBM cells [205,206]. The addition of Monastrol to the combination of ionizing radiation and UCN-01, a CHK1 inhibitor, also exacerbated cell death in cell lines of different types of cancer [207]. SCH2047069, a potent KSP inhibitor, led to mitotic arrest in several cancer cell types. In some cell lines mitotic arrest occurred 4 h after drug exposure, while in others, it took 24 h. Nonetheless, the mechanism behind this difference was not determined. In vivo, SCH2047069 also showed antitumor effects in several cancer types. These effects were time- and dose-dependent. In addition, SCH2047069 was found to promote the antitumoral effects of Paclitaxel, Gemcitabine, a nucleoside analog, and Vincristine [208]. TNBC stem cells expressing ID1 are associated with chemoresistance. For instance, treatment with drugs such as Paclitaxel leads to the enrichment of ID positive TNBC stem cells. Furthermore, in TNBC stem cells, ID proteins are involved in several processes associated with cancer progression and aggressiveness such as proliferation, and metastasis. These processes seem to be regulated by the *Id-Kif11*/*Aurka* axis. ID1 seems to regulate KIF11 expression since ID1 knockdown led to decreased KSP mRNA levels, while KIF11 knockdown led to reduction in CDK1 and Aurora A gene expression. Treatment with Ispinesib promoted death of ID positive cells while increasing the sensitivity of this subpopulation to Paclitaxel. In addition, expression of both ID1 and KIF11 was reduced after exposure to the combination of Ispinesib and Paclitaxel [150]. The combination of Paclitaxel and STLC increased mitotic arrest duration and cells undergoing mitotic slippage in different cell lines. It also slightly increased cell death when compared to STLC alone while marginally improving clonogenicity [209]. A combinatorial approach using PEGylated cationic liposomes containing a KSP siRNA and Paclitaxel in ovarian cancer cells resistant to KSP inhibition showed synergistic effects and overcame KSP inhibition resistance. It seems that the mechanism behind the Paclitaxel-mediated sensitization to KSP inhibitors is that Paclitaxel stabilizes microtubules which might prevent KIF15 activity [210]. Co-silencing of VEGF and KSP with siRNAs in HCC cells reduced migration and cell invasion while increasing apoptosis when compared to both siRNAs alone possibly by decreasing Cyclin D1, BCL-2 and Survivin expression. Moreover, VEGF-siRNA was also shown to reduce KSP expression which suggests that VEGF plays a role in KSP expression regulation [211]. Methotrexate-conjugated polyplexes with KSP siRNA showed increased anticancer effects both in vitro and in vivo cervix carcinoma models [212]. Nanoparticle with a KSP siRNA and Pretubulysin, a microtubule targeting agent, resulted in increased anticancer activity in epidermal growth factor receptor (EGFR) overexpressing cancer cells when compared to both KSP siRNA and Pretubulysin alone [213]. Ispinesib in combination with Elacridar, an inhibitor of the efflux transporters permeability glycoprotein (P-GP) and breast cancer resistance protein (BCRP), reduced tumor growth and increased survival in a GBM mouse model. Ispinesib was found to be a substrate of both P-GP and BCRP and thus their inhibition increases its accumulation in the brain improving its anticancer activity [214]. The combination of Chlorpromazine, which inhibits KSP, with Pentamidine, an antimicrobial drug, showed synergistic anticancer effect by reducing cell growth both in vitro and in vivo. It is suggested that the synergistic effect can be explained by the fact that Pentamidine causes a delay in anaphase leading to impaired chromosome segregation and postmitotic DNA bridges in cells that overcome Chlorpromazine-induced mitotic arrest and consequent cell death. Furthermore, both compounds in combination with microtubule targeting agents also showed synergistic effects in vitro as well as in vivo [215]. In MM, synergistic effects were observed for the combination of Filanesib with Pomalidomide and Dexamethasone both in vitro and in vivo. This combination was shown to increase the activation of the proapoptotic protein BCL-2-associated X protein (BAX) which is associated with Filanesib sensitivity [216]. Furthermore, SB743921 led to MM cell death by repressing the NF-κB pathway. SB743921 administration was also shown to overcome Bortezomib resistance since in Bortezomib-resistant MM cells SB743921 combined with Bortezomib led to increased cell death [217]. On the other hand, addition of Monastrol to Bortezomib treatment reduced Bortezomib-induced neurotoxicity in vivo while it did not interfere with Bortezomib anticancer activity in vitro [218]. A 16 h pre-exposure of CRC cells to Ispinesib followed by exposure to SCH1473759, MK-0457, AT-9283, all inhibitors of Aurora A and Aurora B, or Barasertib, an Aurora B inhibitor, reduced the time these inhibitors needed to increase the number of cells with >4 N DNA content from 24 h to only 4 h. This might be explained by the fact that these inhibitors accelerate exit from Ispinesib-induced mitotic arrest. However, concurrent exposure to Ispinesib and SCH1473759 did not lead to this reduction [219]. Moreover, in HeLa cells, SB743921 was shown to synergize with Alisertib, an Aurora A and B inhibitor, BX795, which targets 3-phosphoinositide-dependent kinase 1, and MK-5108, an Aurora A targeting drug. In a subset of SB743921-resistant cell lines, overexpression of KIF15 was observed, while KIF15 depletion sensitized these cells to SB743921. In addition, targeting of Aurora A was also shown to overcome SB743921 resistance [220]. An antibody-drug conjugate (ADC) with SB743921 and Trastuzumab, an HER2 monoclonal antibody, showed similar results to T-DM1, a commercial ADC, both in vivo and in vitro. Moreover, in vivo it showed less toxicity than T-DM1 making it a promising therapeutic approach [221]. In hematologic malignancies, an ADC comprising an interleukin 3 receptor α (ILR3α) antibody and a KSP inhibitor was explored. This ILR3α-ADC was created to try to limit KSP inhibition to cancer cells and to improve KSP inhibitors therapeutic window. In vitro, the ADC showed higher anticancer effects in ILR3α expressing cells than ones not expressing ILR3α. In vivo, ILR3α-ADC led to increased survival and repressed tumor growth while being well-tolerated [222]. Furthermore, a phase I clinical trial is currently undergoing for this ADC (NCT06034275). A different ADC consisting of a tumor necrosis factor-like weak inducer of apoptosis receptor (TWEAKR) monoclonal antibody and a KSP inhibitor was tested both in vitro and in vivo. In vitro, the KSP inhibitor was shown to promote immunogenic cell death, while in vivo, TWEAKR-ADC showed no antitumor activity in immunocompromised mice but in immunocompetent ones it reduced tumor growth and increased the presence of CD45^+^ leukocytes and CD4^+^ and CD8^+^ T lymphocytes in tumor samples demonstrating it induces an anticancer immune response [223]. In NB cells, the KSP inhibitor 4SC-205 led to mitotic arrest and consequently apoptosis, while reduction in tumor growth was observed in vivo. Furthermore, in a NB liver metastasis mouse model this drug delayed metastatic outgrowth and increased survival. When combined with Cisplatin, Doxorubicin, Topotecan (a Top1 inhibitor), Selumetinib (a MEK1/2 inhibitor), or the Anaplastic lymphoma kinase inhibitors Ceritinib and Lorlatinib, mostly addictive effects were observed [144]. A phase I study assessing 4SC-205 administration in patients with advanced malignancies is currently ongoing (NCT01065025). In TNBC cell lines, the combination of Vinblastine with Monastrol or Ispinesib displayed synergistic anticancer activity by increasing mitotic arrest and consequently apoptosis. In vivo, Ispinesib and Vinblastine synergistically reduced tumor growth [224]. In GBM, Ispinesib was shown to synergize with inhibitors of WNK lysine deficient protein kinase 3 inhibitor, RIO kinase 1, MYB proto-oncogene transcription factor or Cathepsin F [225]. In BC cell lines, STLC and Monastrol were found to be more effective in estrogen receptor-positive cells than in negative ones. Moreover, when combined with Fulvestrant, an estrogen receptor inhibitor, the IC_50_ for each drug increased, while combination with E2 led to the opposite effect. This may be explained by the fact that Fulvestrant action leads to decreased expression of KSP, while the contrary happens with E2 [226]. S(MeO)TLC was shown to suppress tumor growth both in vitro and in vivo, in Gemcitabine-resistant bladder cancer. Addition of Gemcitabine did not significantly increase the anticancer effects observed with S(MeO)TLC alone [227]. In malignant peripheral nerve sheath tumor, Ispinesib and Filanesib were shown to inhibit cell proliferation and induce cell death. Moreover, Filanesib in combination with JQ1, a bromodomain-containing protein 4 inhibitor, showed synergistic anticancer effects, while KIF15 knockdown increases sensitivity to KSP inhibitors and also to the combination of Filanesib with JQ1 [228]. HR22C16, a selective KSP inhibitor, was demonstrated to sensitize the lung cancer cell line H1299 to TRAIL by downregulating survivin, BCL-2 and X-linked inhibitor of apoptosis and by repressing NF-κB [229]. Moreover, in vitro, KSP inhibition combined with the administration of Cisplatin was shown to increase Cisplatin-induced ototoxicity [230]. Several combinatorial approaches have shown synergistic effects. Interestingly, synergistic effects were observed when KSP inhibitors were combined with drugs targeting mitosis such as inhibitors of Aurora A and B and microtubule-targeting agent such as Vinblastine and Paclitaxel. However, these results might not translate when treating cancer patients, so it is important to test these combinations in clinical trials.

### 6.3. Clinical Trials

Some of these drugs have already been tested in clinical trials with Ispinesib and Filanesib being the most evaluated KSP inhibitors. In this section, the clinical trials results will be discussed (Table 3).

For instance, in patients with solid tumors MK-0731 led to prolonged disease stability with tolerable toxicity profile [10]. In a phase I clinical trial with patients with advanced solid tumors, Litronesib showed no objective tumor responses but the recommended dose for the combination with granulocyte colony-stimulating factor was determined [231]. A different study with Litronesib showed similar results demonstrating this drug does not show clinical efficacy [232]. Similarly, AZD4877 in patients with solid and lymphoid malignancies showed manageable toxicity with no objective responses observed, while in patients with recurrent advanced urothelial cancer, limited clinical efficacy was reported [8,9,233]. Additionally, a clinical trial with AZD4877 in patients with refractory acute myeloid leukemia was terminated early due to lack of efficacy [234]. EMD 534085 had limited activity although it showed manageable safety profile [235]. Contrarily to the other drugs, one partial response was observed with SB-743921, while stable disease was reported for several patients. In addition, the toxicity profile was deemed manageable [236]. Additionally, in patients with relapsed or refractory lymphoma, addition of granulocyte stimulating factor to SB-743921 treatment increased the maximum tolerated dose and clinical activity of this inhibitor [237].

Ispinesib has also been investigated in clinical trials and in patients with solid tumors the maximum tolerated dose was achieved and it was well tolerated [238,239]. Nonetheless, in patients with androgen-independent, and mostly Docetaxel-resistant, prostate cancer it showed no efficacy [240]. In HCC, treatment with Ispinesib was explored and was mostly well tolerated but with no clear clinical benefit [241]. Similarly, Ispinesib in patients with metastatic or recurrent malignant melanoma was tolerable but showed no objective responses [242]. Furthermore, in patients with advanced renal cell cancer it also did not show clinical activity [243]. This drug was also combined with Docetaxel and at the concentrations used in the clinical trial showed no improvement of anticancer activity but with manageable toxicity [244]. In a phase 2 study, administration of Ispinesib in recurrent or metastatic head and neck squamous cell carcinoma patients led to an overall survival of 3.5 months, however no anticancer activity was observed and the study was terminated early [245].

Additionally, Filanesib also demonstrated mostly no clinical benefit in clinical trials, as monotherapy [246,247]. However, in patients with refractory MM, clinical activity with manageable toxicity was reported [248]. Moreover, addition of Filanesib to the combination of Pomalidomide, modulator of the E3 ubiquitin ligase component Cereblon, and Dexamethasone, a glucocorticoid agonist, in refractory MM patients promoted their activity although with increased toxicity [249]. Similarly, Filanesib combined with Bortezomib, a proteasome inhibitor, and Dexamethasone showed promising activity and was deemed safe [250,251]. A phase I study exploring the combination of Filanesib, Carfilzomib, a proteasome inhibitor, and Dexamethasone showed that, in patients with MM, the regimen was safe but with limited efficacy [252].

A phase I trial exploring a lipid nanoparticle containing siRNAs for VEGF and KSP in patients with different cancer types with liver involvement showed that this approach had antitumoral effects with a complete response being observed for a patient with endometrial cancer and multiple hepatic metastases. This approach was also deemed tolerable [253]. The results in clinical trials with KSP inhibitors in monotherapy have been disappointing for the most part. However, some combinatorial approaches have improved antitumor activity. Thus, these drugs can still be potential treatment options and should still be investigated in combination with other drugs.

**Table 3:** Clinical trials exploring KSP inhibitors in the treatment of cancer.

Drug	Disease	Intervention	Phase	Results	NCT/References
Ispinesib	Metastatic or unresectable solid tumors or Hodgkin’s or non-Hodgkin’s lymphoma	Ispinesib	Phase I	Terminated with no published results	NCT00101244
	Acute leukemia, chronic myelogenous leukemia, or advanced myelodysplastic syndromes	Ispinesib	Phase I	Completed with no results	NCT00098826
	Pediatric solid tumors	Ispinesib	Phase I	The recommended phase II dose was weekly 9 mg/m^2^ for 3 consecutive weeks	NCT00363272 [239]
	Metastatic breast cancer	Ispinesib	Phase I/II	The trial was terminated	NCT00607841
	Platinum-Taxane-refractory or resistant relapsed ovarian cancer	Ispinesib	Phase II	Completed with no results	NCT00097409
	Advanced or metastatic NSCL cancer	Ispinesib	Phase II	Completed with no results	NCT00085813
	Androgen-independent prostate cancer previously treated with taxanes	Ispinesib	Phase II	Ispinesib showed lack of efficacy possibly due to low expression of KSP in the population of the study	NCT00096499 [240]
	Metastatic hepatocellular carcinoma	Ispinesib	Phase II	Ispinesib was well tolerated but no clear clinical benefit was found	NCT00095992 [241]
	Metastatic or recurrent malignant melanoma	Ispinesib	Phase II	No objective responses were observed but Ispinesib was well tolerated	NCT00095953 [242]
	Advanced renal cell cancer	Ispinesib	Phase II	Ispinesib showed limited cytotoxic effect at the dose used.	NCT00354250 [243]
	R/M HNSCC	Ispinesib	Phase II	No antitumor activity observed	NCT00095628 [245]
	Advanced or metastatic breast cancer	Ispinesib	Phase II	In total, 4 patients responded to treatment. Nonetheless the duration of these responses was short (from 6.9 to 19 weeks). Moreover, none of the 50 patients completed treatment mostly due to disease progression	NCT00089973
	Advanced or metastatic colorectal cancer	Weekly Ispinesib or every three weeks	Phase II	No objective responses observed. Administration of Ispinesib at 7 mg/m^2^ over 1 h on days 1, 8, and 15 (repeated every 28 days) led to a progression-free survival (PFS) of 7 weeks and an overall survival (OS) of 3.6 months, while Ispinesib at 18 mg/m^2^ over 1 h on day 1 (repeated every 21 days) led to a PFS of 5.3 weeks and an OS of 4.5 months. Adverse events were common but tolerable since only one patient withdrew due to adverse events	NCT00103311
	Solid tumors	Ispinesib with Capecitabine	Phase I	No results published	NCT00119171
	Solid tumors	Ispinesib with Carboplatin	Phase I	Completed with no results	NCT00136578
	Advanced solid tumors	Ispinesib with Docetaxel	Phase I	The combinatorial approach was tolerable, and the maximum tolerated dose (MTD) was found (10 mg/m^2^ of Ispinesib and 60 mg/m^2^ of docetaxel)	NCT00169520 [244]
Filanesib	Advanced myeloid leukemias	Filanesib	Phase I	A MTD of 4.5 mg/m^2^ was determined, however low clinical activity was observed	NCT00637052 [247]
	Advanced solid tumors	Filanesib with or without Filgrastim	Phase I	Filanesib led to 7 out of 39 stable disease responses and was well tolerated	NCT00462358 [246]
	Relapsed/refractory multiple myeloma	Filanesib, Carfilzomib, and Dexamethasone	Phase I	The combinatorial approach showed manageable toxicity but with marginal efficacy	NCT01372540 [252]
	Relapsed/ refractory t(11;14) and 1q21 gain multiple myeloma	Filanesib in combination with Bortezomib and Dexamethasone	Phase I	An ORR of 39% and a median duration of response of 18.0 months were observed for this combination	NCT01248923 [251]
	Recurrent/refractory multiple myeloma	Filanesib with prophylactic Filgastrim, Bortezomib, and Dexamethasone	Phase I	Manageable toxicity and durable responses were observed for this combination	NCT01248923 [250]
	Relapsed/refractory multiple myeloma	Filanesib plus Filgrastim with or without Dexamethasone	Phase I/II	The MTD for Filanesib was 1.50 mg/m^2^/day. Filanesib alone led to an overall response rate (ORR) of 16%, clinical benefit rate (CBR) of 23% and OS of 19.0 months, while Filanesib with Dexamethasone showed an ORR of 15%, CBR of 20% and OS of 10.7 months	NCT00821249 [248]
	Relapsed/refractory multiple myeloma	Filanesib, combined with Pomalidomide and Dexamethasone	Phase Ib/II	The combinatorial approach led to 51% of patients achieving partial response, PFS of 7 months and OS of 19 months. A better ORR (62% vs. 17%) and PFS (9 vs. 2 months) were observed for patients with low serum levels of alpha 1 acid glycoprotein at baseline	NCT02384083 [249]
	Relapsed/refractory multiple myeloma	Filanesib with Filgrastim and G-CSF	Phase II	Completed with no published results	NCT02092922
	Advanced multiple myeloma	Carfilzomib vs. Filanesib and Carfilzomib	Phase II	Completed with no published results	NCT01989325
LNP-formulated RNAi	Cancer patients with liver involvement	RNA interference therapeutic targeting VEGF and KSP	Phase I	The treatment approach was deemed safe with clinical activity	NCT00882180 [253]
VIP943	Advanced CD123+ hematologic malignancies	VIP943 (a CD123-targeting ADC with a KSP inhibitor)		Currently recruiting	NCT06034275
Litronesib	Advanced cancer	Litronesib with or without Pegfilgrastim	Phase I	The main dose-limiting adverse effect was neutropenia as observed for other KSP inhibitors. The study also recommends two regimens for a phase II trial (21-day cycles of 6 mg/m^2^/day of Litronesib plus Pegfilgrastim on days 1, 2, 3 or 8 mg/m^2^/day of Litronesib plus Pegfilgrastim, on days 1, 5, 9)	NCT01214629 and NCT01214642 [232]
	Advanced solid tumors	Litronesib and G-CSF	Phase I	The recommended dose of LY2523355 combined with G-CSF for subsequent trials was 5 mg/m^2^/day, nonetheless, no objective tumor responses were reported	NCT01358019 [231]
	Small-Cell Lung Cancer	Litronesib with or without G-CSF	Phase II	PFS, CBR, and ORR of Litronesib alone were 5.3 weeks, 23.7% and 2.6%, respectively, vs. 6.1 weeks and 26.9% for Litronesib with G-CSF, while the ORR was not calculated since no participants had complete responses or partial responses	NCT01025284
	Ovarian, non-small cell lung, prostate, colorectal, gastroesophageal cancers, and head and neck squamous cell carcinoma	Litronesib with Pegfilgrastim	Phase II	The PFS observed with Litronesib with Pegfilgrastim ranged from 1.25 to 2.3 months for the different types of cancer, while the percentage of patients that achieved CR, PR or stable disease ranged from 16.7% to 50%.	NCT01059643
	Breast cancer	Litronesib with Pegfilgrastim or Filgrastim vs. Ixabepilone	Phase II	Litronesib with Pegfilgrastim or Filgrastim led to a PFS, CBR and ORR of 1.71 months 46.2% and 3.8%, respectively, while Ixabepilone showed a PFS, CBR and ORR of 2.76 months 61.5% and 7.7%, respectively	NCT01416389
SB-743921	Relapsed or refractory lymphoma	SB-743921 with or without G-CSF	Phase I	The MTD of SB-743921 was 6 mg/m^2^, while with G-CSF it was 9 mg/m^2^. In total, 4 patients showed partial responses, while stable disease was observed for 19 of the 56 patients analyzed	NCT00343564 [237]
AZD4877	Solid and lymphoid malignancies	AZD4877	Phase I	The treatment was well tolerated but no clear clinical benefit was observed	NCT00471367 [8]
	Advanced solid tumors	AZD4877	Phase I	The regimen was tolerable and the MTD was achieved (30 mg of AZD4877 given as a 1 h iv infusion on days 1, 8, and 15 of a 28 days cycle)	NCT00389389 [9]
	Refractory acute myeloid leukemia	AZD4877	Phase I/II	Terminated due to lack of efficacy	NCT00486265 [234]
	Recurrent advanced urothelial cancer	AZD4877	Phase II	The regimen was well tolerated but with limited efficacy. No further studies are warranted	NCT00661609 [233]
MK-0731	Sollokl, id tumors	MK-0731	Phase I	The MTD for MK-0731 was 17 mg/m^2^/day every 21 days. Treatment was well tolerated leading to stable disease in heavily pretreated patients	NCT00104364 [10]
EMD 534085	Advanced solid tumors or lymphoma	EMD 534085	Phase I	EMD 534085 MTD was 108 mg/m^2^/day. The treatment was well tolerated but with limited activity	[235]
4SC-205	Advanced malignancies	4SC-205	Phase I	Completed with no published results	NCT01065025

### 6.4. Resistance to Treatment

Cancer cells can acquire mutations that can lead to resistance to KSP targeting drugs [254,255,256]. For instance, in colon cancer cell lines, the point mutation T107N in KSP conferred resistance to treatment with STLC, Ispinesib and Filanesib [257]. Additionally, several mutations (R119A, D130A, P131A, L132A, I136A, V210A, Y211A, L214A and E215A) confer resistance to inhibition with Monastrol, while STLC resistance was observed for R119A, D130A, and L214A mutants [258,259,260]. Furthermore, D130A mutants were also shown to be resistant to Filanesib and Ispinesib while the mutation L214A only conferred resistance to Filanesib [261]. The point mutation Y104C, has been associated with resistance to BRD9876, while D130V mutants were more sensitive to this inhibitor [262]. A133D and D130V mutants have been shown to confer resistance to SB743921 while I299F and A356T mutants are resistant to GSK-1 [263,264].

Even though, inhibition of KSP can lead to cancer cell death cells have different pathways that can compensate for KSP activity which can translate into resistance to KSP targeting. For instance, during prophase, KSP activity in centrosome separation can be compensated by nuclear envelope-associated dynein activity [45]. KIF15 overexpression can also confer resistance to KSP inhibition probably by compensating for some KSP functions [60,220,265]. Nonetheless, in HeLa cells, instead of KIF15 overexpression, it was the increase in spindle microtubule bundling that promoted KIF15 compensation of KSP activity in resistant cells [266]. Moreover, it was recently proposed that PRC1 plays a role in the KIF15 compensation of KSP activity when KSP is inhibited. PRC1 promotes the binding of KIF15 to microtubules, and its inhibition leads to reduced spindle bipolarity in cells resistant to KSP inhibitors. Additionally, overexpression of PRC1 also induces KSP inhibition resistance [267]. HSP70 has also been reported to promote resistance to KSP targeting drugs. HSP70 is involved in the association of KSP with TPX2 and consequently in the regulation of KSP distribution and function and its inhibition enhanced the cytotoxic effects of KSP inhibitors [53]. In MM, the inhibition of KSP led to the upregulation of HSP70 through the PI3K/AKT pathway. Moreover, co-targeting of KSP and farnesyltransferase showed synergistic effects by interfering with the PI3K/AKT pathway [268]. High mRNA expression of TPX2, MYBL2 and Aurora A have also been found to be associated with cancer cells resistance to KSP inhibition. Accordingly, inhibition of Aurora A, MYBL2 or TPX2 overcame this resistance [269].

In GBM cells, resistance to Ispinesib treatment emerged due to the activation of signal transducer and activator of transcription 3, mediated by SRC and EGFR, which inhibits apoptosis [270]. Furthermore, EGF signaling promotes premature centrosome separation and prevents mitotic arrest upon KSP inhibition avoiding the necessity of KSP for mitosis [271]. Similarly, depletion of Tiam1 or its downstream targets PAK1/2, which oppose centrosome separation, has been shown to prevent monopolar arrest caused by KSP inhibition [25,26]. In bladder cancer cell lines, knockdown of p63 or its downstream target c-MYC was shown to confer resistance to AZD4877. In accordance, AZD4877-resistant cells had lower expression of p63 [272]. Moreover, in relapsed/refractory MM patients, higher levels of alpha 1-acid glycoprotein were associated with less sensitivity to Filanesib [248].

Monastrol-resistant breast cancer cells were reported to downregulate the CircRNA-MTO1. CircRNA-MTO1 acts as a CeRNA binding to TRAF4 and thus preventing KSP gene expression. Consequently, increasing the expression of CircRNA-MTO1 overcame Monastrol resistance [273]. A different study demonstrated that sensitivity to Monastrol is dependent on survivin expression, as survivin upregulation reduces mitotic slippage, and thus Monastrol efficacy [274].

In addition, cancer cells can export small molecule inhibitors, reducing their efficacy. For instance, as previously stated, Ispinesib is a substrate of the efflux transporters P-GP and BCRP. At the blood–brain barrier these transporters restrict Ispinesib delivery limiting its anticancer effects [214,275]. Nonetheless, Ispinesib-resistant cells seem to form less aggressive tumors which was corroborated in a Ispinesib-resistant GBM mouse model where resistant mice showed increased survival [225].

Several mechanisms of resistance to KSP inhibition have been reported and it is possible that co-targeting of KSP and some of these pathways can be a useful strategy in cancer treatment. The type of point mutations that can occur in cancer cells should also be taken into account since they can be compensated by administrating a different KSP inhibitor.

### 6.5. Overcoming Resistance by Combining KSP Inhibitors with Anti-Apoptotic Protein Targeting

Given the results from clinical trials, where KSP inhibitors as standalone treatments showed limited or no efficacy, along with the development of resistance, targeting KSP as a monotherapy does not appear to be a viable strategy for cancer treatment.

However, combinatory strategies involving KSP inhibitors have shown promising results, suggesting that combining KSP inhibition with other therapeutic approaches could enhance its efficacy. Since cancer cells often manipulate pro-apoptotic and anti-apoptotic proteins to evade cell death, combining KSP inhibitors with drugs targeting anti-apoptotic proteins may provide a more effective treatment strategy, potentially overcoming resistance mechanisms and improving patient outcomes. For instance, activation of SAC followed by mitotic slippage caused by KSP inhibition leads to BAX activation which initiates apoptosis [276]. Monastrol addition in HeLa cells induced mitotic arrest leading to apoptosis through the mitochondrial/intrinsic pathway [277]. In this pathway, mitochondrial outer membrane permeabilization occurs by inhibition of pro-survival proteins, including MCL-1, BCL-2 and BCL-extra-large (BCL-xL) [277]. BCL-2 and BCL-xL have been reported to sequester BAX preventing apoptosis induction [278]. Moreover, KSP-IA, a KSP inhibitor, has been shown to promote apoptosis by BAX activation [279]. Similarly, the KSP inhibitor KPYB10602-induced apoptosis led to increased BAX/BCL-2 ratio [198].

Moreover, as previously stated, hematologic cells seem to rely on MCL-1 to prevent apoptosis. Since MCL-1 is a less stable survival protein, it might make these cells more sensitive to KSP inhibition. On the other hand, it is suggested that cells that rely on BCL-2 or BCL-xL, which are more stable antiapoptotic proteins, can prevent cells arrested in mitosis of undergoing apoptosis. This can be explained by the fact that more stable antiapoptotic proteins will not be degraded fast enough to reach the cell death threshold. Moreover, the clinical half-lives of KSP inhibitors usually range from 28 to 50 h and thus cells can maintain a prosurvival signal long enough for these inhibitors levels to fall below therapeutic levels. Then cells can correct the defects caused by the inhibition of KSP and normally divide [169]. In a lung cancer cell line, Monastrol could not induce apoptosis but led to mitotic slippage resulting in tetraploid cells arrested in G_1_. It was found that the prevention of apoptosis induction was caused by the overexpression of BCL-xL and that its depletion sensitized the cells to Monastrol. Moreover, Fas receptor, a death receptor involved in the promotion of apoptosis, was also overexpressed after Monastrol exposure. Combining Monastrol administration with a Fas agonist led to synergistic antitumor effects [280]. In a promyeoloblast cell, overexpression of BCL-2 was also shown to protect against KSP inhibition with EMD 534085 [281].

In several cancer cell lines, the combination of EMD 534085 with Navitoclax, an inhibitor of BCL-xL and BCL-2, increased cell death when compared with both drugs alone [282]. Recently, in oral squamous cell carcinoma cell lines, we showed that Ispinesib combined with Navitoclax led to synergistic effects by enhancing the cytotoxic effects of Ispinesib alone. To ensure that the observed effects were due to the combinatorial approach rather than the specific drugs used, we also tested a combination of Filanesib with ABT-737. This combination produced results similar to those obtained with Ispinesib and Navitoclax, further supporting the potential of the combinatorial strategy [283].

## 7. Conclusions

KSP plays a pivotal role in mitosis by orchestrating bipolar spindle formation and ensuring accurate chromosome segregation, processes essential for maintaining genomic stability. Its consistent overexpression in a variety of cancers, coupled with its critical function in sustaining proliferative signaling, underscores its value as a promising and specific therapeutic target in cancer. The inhibition of KSP has already demonstrated anti-tumor activity in preclinical and early clinical studies, particularly through the induction of mitotic arrest and apoptosis in rapidly dividing cancer cells.

To advance the clinical utility of KSP-targeted therapies, future research should prioritize uncovering the precise molecular mechanisms regulating its activity, including post-translational modifications and interactions with mitotic regulators. Equally important is the investigation of resistance mechanisms, such as point mutations, compensatory pathways (e.g., KIF15 and PRC1 overexpression), and the activity of anti-apoptotic proteins (e.g., BCL-2, MCL-1, BCL-xL), which limit the efficacy of current KSP inhibitors. Combinatorial strategies, including KSP inhibitors with chemotherapeutic agents, targeted therapies, or anti-apoptotic protein inhibitors, have shown promise in overcoming these resistance mechanisms, enhancing mitotic arrest, promoting apoptosis, and improving antitumor efficacy.

Importantly, the development of biomarkers for patient stratification will be critical to maximize the therapeutic potential of KSP-targeted treatments. Identifying molecular signatures that predict response or resistance (e.g., TPX2, MYBL2, Aurora A) can guide patient selection, optimize combinatorial strategies, and enable a more personalized approach to therapy. Incorporating these insights into clinical practice may enhance the efficacy and safety of KSP inhibitors and support precision oncology initiatives. While KSP may also participate in other physiological processes, such as neuronal function and intracellular transport, its oncogenic relevance makes it a uniquely attractive candidate for selective cancer intervention. Continued efforts to develop potent, specific, and well-tolerated KSP inhibitors could significantly contribute to next-generation anti-mitotic strategies in oncology.

## Figures and Tables

**Table 2:** KSP expression according to UALCAN using CPTAC samples. The data were retrieved from the UALCAN web resource (http://ualcan.path.uab.edu/ (accessed on 3 August 2025)), through the analysis of proteomics data from Clinical Proteomic Tumor Analysis Consortium (CPTAC).

Organ	Cancer Type	Expression
Ovaries	Ovarian cancer	Upregulated
Liver	Hepatocellular carcinoma	Upregulated
Head and neck	Head and neck squamous cell carcinoma	Upregulated
Breast	Breast cancer	Upregulated
Lung	Lung adenocarcinoma	Upregulated
	Lung squamous cell carcinoma	Upregulated
Endometrium	Uterine corpus endometrial carcinoma	Upregulated
Brain and CNS	Glioblastoma multiforme	Upregulated
Pancreas	Pancreatic adenocarcinoma	Upregulated
Colon	Colon cancer	Upregulated
Kidney	Clear cell renal cell carcinoma	Downregulated

## Data Availability

Not applicable.

## Abbreviations

The following abbreviations are used in this manuscript.

γTuRCs	γ-tubulin ring complexes
ADC	Antibody-drug conjugate
APC/C	Anaphase promoting complex/cyclosome
ATP	Adenosine triphosphate
AURKA	Aurora kinase A
BAX	BCL-2-associated X protein
BC	Breast cancer
BCL-2	B-cell lymphoma 2
BCL-xL	BCL-extra-large
BCRP	Breast cancer resistance protein
cAMP	Cyclic adenosine monophosphate
CBR	Clinical benefit rate
CDH1	CDC20-homolog 1
CDK	Cyclin-dependent kinase
CeRNA	Competing endogenous RNA
CHK1	Checkpoint kinase 1
CircRNA	Circular RNA
CLIP-170	Cytoplasmic linker protein 170
CPTAC	Clinical Proteomic Tumor Analysis Consortium
CRC	Colorectal cancer
DLG1	Disks large homolog 1
DNA	Deoxyribonucleic acid
EGF	Epidermal growth factor
EGFR	Epidermal growth factor receptor
EML4–ALK V3	Echinoderm microtubule-associated protein-like 4-anaplastic lymphoma kinase variant 3
FAK	Focal adhesion kinase
FBXO30	F-box only protein 30
GBM	Glioblastoma
HCC	Hepatocellular carcinoma
HDAC1	Histone deacetylase 1
HER2	Human epidermal growth factor receptor 2
HNSCC	Head and neck squamous cell carcinoma
HPV	Human Papillomavirus
HSP70	Heat shock protein 70
ID	Half-maximal inhibitory concentration
IC50	Inhibitor of differentiation
ILR3α	Interleukin 3 receptor α
JNK	c-Jun N-terminal kinase
KAT5	Lysine acetyltransferase 5
KIF	Kinesin family member
KSP	Kinesin spindle protein
Lis1	Lissencephaly-1
LncRNA	Long non-coding RNAs
Lys	Lysine
MCAK	Mitotic centromere-associated kinesin
MG53	Mitsugumin 53
MM	Multiple myeloma
MCL-1	Myeloid cell leukemia 1
miR	MicroRNA
MMP-9	Matrix metalloproteinase 9
mRNA	Messenger RNA
MTD	Maximum tolerated dose
MYBL2	MYB proto-oncogene like 2
NAT10	N-acetyltransferase 10
NB	Neuroblastoma
Nek	NIMA-related kinase
NF-κB	Nuclear factor-κB
NuMA	Nuclear mitotic apparatus
ORR	Overall response rate
OS	Overall survival
P-GP	Permeability glycoprotein
PAK	P21-activated kinase
PDAC	Pancreatic ductal adenocarcinoma
PI3K	Phosphoinositide 3-kinase
PLK1	Polo-like kinase 1
PFS	Progression free survival
PP2A	Protein phosphatase 2A
PRC1	Protein regulator of cytokinesis 1
PTEN	Phosphatase and tensin homolog
RNF20/40	RING finger 20/40
SAC	Spindle assembly checkpoint
Ser	Serine
SFKs	Src family kinases
siRNA	Small interfering RNA
Smurf2	SMAD-specific E3 ubiquitin protein ligase 2
S(MeO)TLC	S-(methoxytrityl)-L-cysteine
SREBP2	Sterol regulatory element-binding protein 2
STLC	S-trityl-L-cysteine
SYVN1	Synoviolin 1
Thr	Threonine
TNBC	Triple negative breast cancer
Top	Topoisomerase
TPX2	Targeting protein for Xklp2
TRAF4	Tumor necrosis factor-receptor associated factor 4
TRIM	Tripartite motif
TWEAKR	Tumor necrosis factor-like weak inducer of apoptosis receptor
Tyr	Tyrosine
UALCAN	University of Alabama at Birmingham cancer data analysis portal
Ufm1	Ubiquitin-fold modifier 1
UHRF1	Ubiquitin-like with PHD and RING finger domains 1
VCP	Valosin-containing protein
VEGF	Vascular endothelial growth factor
VPS28	Vacuolar protein sorting-associated protein 28
